# A new early monofenestratan pterosaur from the Mörnsheim Formation of southern Germany

**DOI:** 10.7717/peerj.21204

**Published:** 2026-05-11

**Authors:** David W.E. Hone

**Affiliations:** School of Biological and Behavioural Sciences, Queen Mary University of London, London, United Kingdom

**Keywords:** Pterosauria, Solnhofen, Non-pterodactyloid, Darwinoptera

## Abstract

A number of new early monofenestratan pterosaurs have recently been described from the Late Jurassic Mörnsheim Formation of Southern Germany, greatly expanding our knowledge of the diversity and evolution of this group. Here, another new taxon from this grade is described and named from the formation: *Laueropterus vitriolus* gen. et sp. nov. The animal is represented by a near complete and well-preserved adult individual that is mostly disarticulated. The species has a large wingspan compared to other members of the grade of c. one m. In a series of limestone beds with distinct preservation and taphonomy compared to the ‘traditional’ Solnhofen archipelago, the animal provides increasing evidence of a later fauna in the region that is dominated by early monofenestratans.

## Introduction

For nearly two and a half centuries, the Solnhofen region of southern Germany has been producing important specimens of pterosaurs from various limestone fossil beds. These include representatives of numerous taxa including early-branching forms (non-monofenestratans, such as *Anurognathus, Scaphognathus*, *Rhamphorhynchus*— Wellnhofer, 1975), “intermediate” early monofenestratans (*e.g.*, *Propterodactylus*, Spindler, 2024; *Skiphosoura*, Hone et al., 2024; *Makrodactylus*, Hone et al., 2025), and members of the derived clade the pterodactyloids (*e.g.*, *Pterodactylus, Gnathosaurus, Ctenochasma* - Wellnhofer, 1970).

The early monofenestratans are a relatively new discovery in pterosaur evolution first being recognized in 2010 (Lü et al., 2010). Various analyses have recovered these taxa as both a clade (Martin-Silverstone et al., 2024) and grade (Hone et al., 2024) between the non-monofenetratans and the pterodactyloids. Some of the most derived of these taxa have been named as pterodactyliforms and represent clade of derived monofenestratans and the pterodactyloids (Andres, Clark & Xu, 2014).

Both the quantity (*e.g.*, Bennett, 1995) and quality (*e.g.*, Frey et al., 2003) of Solnhofen specimens have been important in studying the anatomy, biology and evolution of the pterosaurs. The region remains a major source of data on pterosaurs, with new finds of well-known taxa (*e.g.*, Augustin et al., 2022) and indeed new species (*e.g.*, *Balaenognathus*, Martill et al., 2023) still being regularly recovered, and new information arising from old specimens (*e.g.*, Jäger et al., 2018).

In recent years, excavation of quarries of the previously under-explored Mörnsheim Formation has been important in providing a number of new and important vertebrate fossils (*e.g.*, Rauhut, Heyng & Leonhardt, 2011; Rauhut, Tischlinger & Foth, 2019; Herrera, Aiglstorfer & Bronzati, 2021) and in particular, pterosaurs (*e.g.*, Hone et al., 2023; Hone et al., 2024; Hone et al., 2025). Many of these finds have already been identified as new taxa, and it is clear that the Mörnsheim likely represents not just a different period of time to the better-known local formations, but was also a fundamentally different environment, as shown by the unusual preservation of specimens that are complete but fully disarticulated (Hone et al., 2023), and that the pterosaurs are typically those associated with terrestrial rather than marine ecosystems (Hone et al., 2025).

**Figure 1:**
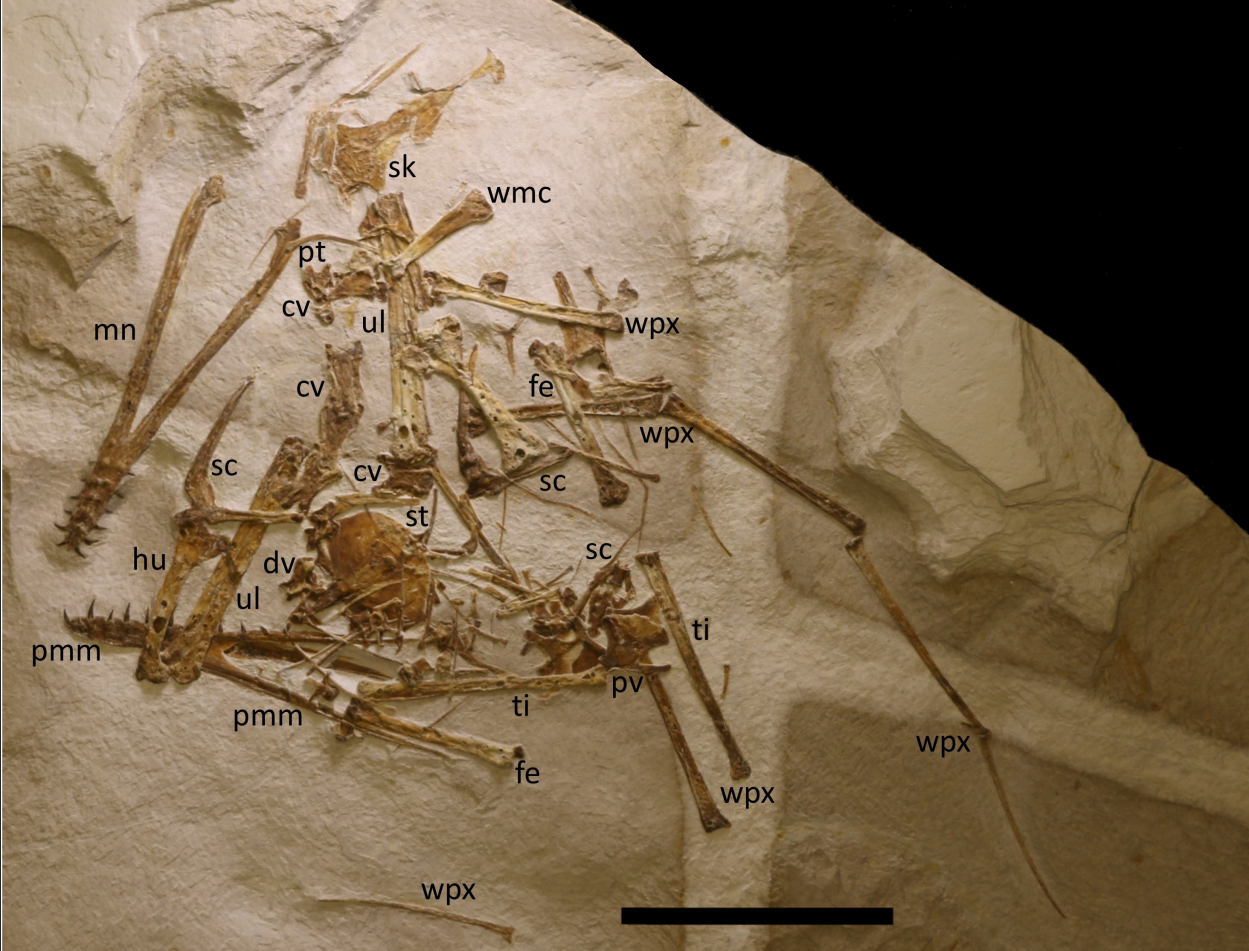
Complete specimen of LF 6268. The holotype specimen seen under natural light. Major elements are labeled. Abbreviations as follows here and in subsequent figures: ca, caudal vertebra; cp, carpal; cv, cervical vertebrae; dv, dorsal vertebra; fe, femur; hu, humerus; mn, mandible; mt, metatarsal; ph, phalanges; pmm, premaxilla-maxilla; pp, prepubis; pt, pteroid; pv, pelvic plate; r, rib; sa, sacrum; sc, scapulocoracoid; sk, skull part; st, sternum; ta, tarsals; ti, tibia; ul, ulna; un, ungual; wpx, wing phalanges; wmc, wing metacarpal. Scale bar is 100 mm.

Here a new genus of early (*i.e.,* non-pterodactyloid) monofenestratan pterosaur *Laueropterus vitriolus*, is described with an unusual red tint to the preserved bones, which also have suffered a unique form of taphonomic pitting (Fig. 1).

## Materials & Methods

### Locality information and specimen history

The specimen was found around 2007 in the ‘private section’ of the Schaudiberg Quarry, near the town of Mülheim, Bavaria, Germany. It was found at a site reported as being three m below the “Rhamphodactylus” (Rauhut, 2012)—the layer which also produced *Makrodactylus* (Hone et al., 2025). This is within the Mörnsheim Formation and so this would be Tithonian in age.

The specimen was sold in an unprepared state to Wolfgang Krauss, a prominent collector, who then sold the specimen (still unprepared) to Günther Hoppe from Switzerland. Preparation was performed by Beat Imhof in Switzerland, before it was sold to Martin Görlich. Stabilisation of the fragile specimen was then carried out by Stefan Selzer, and the Lauer Foundation acquired it in January 2024, as LF 6268. All preparation work done on the specimen by both preparators was mechanical with tools (Stefan Selzer, pers. comm., February, 2026).

The specimen is permanently held with the Lauer Foundation for Paleontology, Science and Education in the USA. The mission of the Lauer Foundation is to curate its fossil collection providing the scientific community and other museums with permanent access for the purposes of exhibition, study and education. Public access to type and figured specimens, as well as specimens listed or cited in publications together with other scientifically important specimens is guaranteed.

## Methods

**Figure 2:**
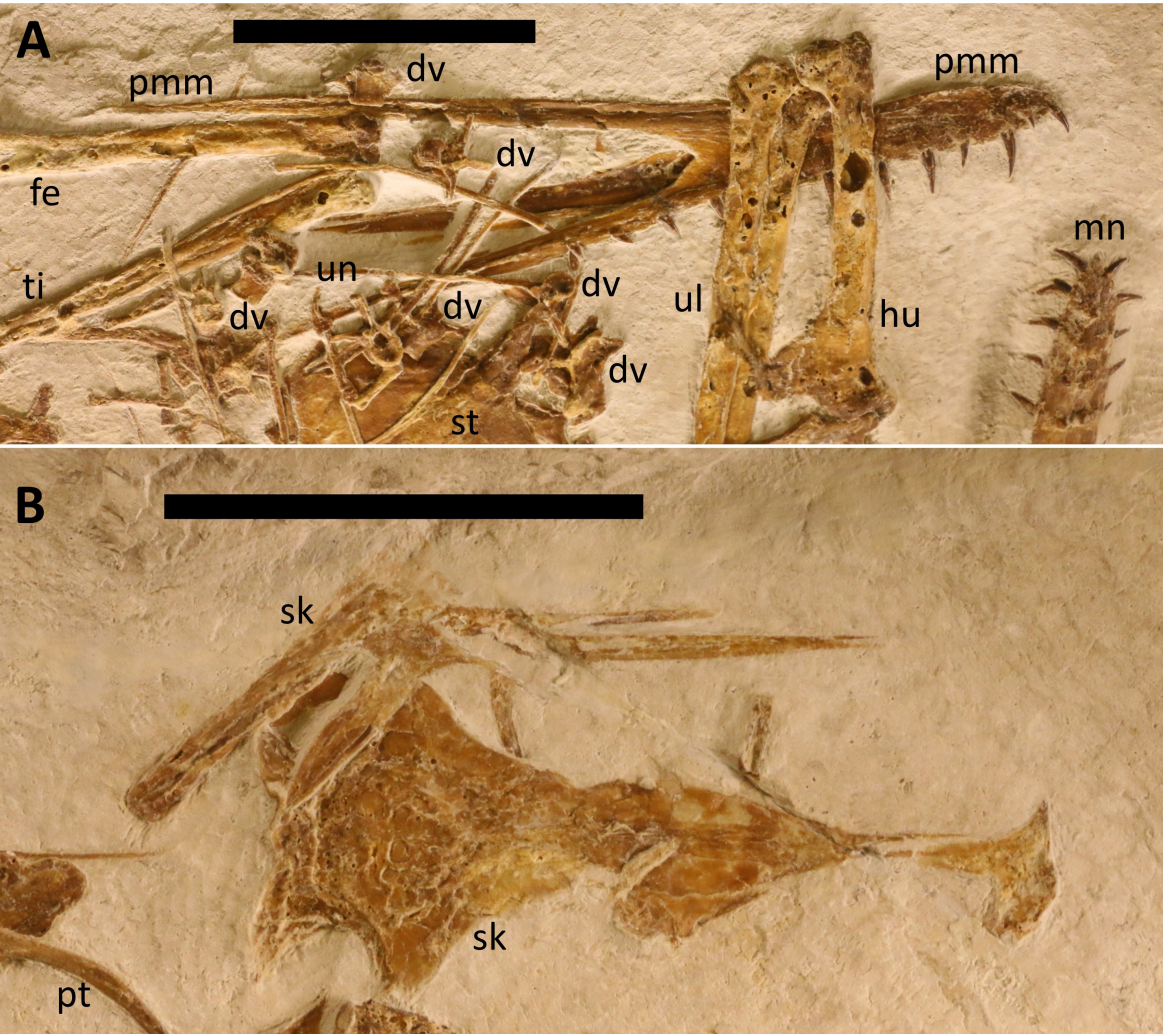
The skull of LF 6268. Major skull elements of LF 6268. (A) Rostrum with the premaxilla-maxilla complex and teeth seen in right lateral view. (B) Skull roof seen in dorsal view with the left quadrate, jugal and quadratojugal in lateral view. Labels as in Fig. 1. Scale bars are 50 mm and 10 mm respectively.

The specimen examined under various UV light regimes (UVA and B and a mixture of the two). This helps to distinguish the bones from the matrix, though this is already simple here, given the contrast between the two, unlike some other specimens from the formation (*e.g.*, see Hone et al., 2025). However, as with other pterosaurs described from the Mörnsheim Formation so far, no soft tissues were observed. As such, all photography presented here and the examination and description of the specimen was carried out under normal lighting (see Fig. 2) using a Canon EOS 70D with a Canon 18–135 mm variable zoom lens. Measurements were taken with a set of digital calipers.

The electronic version of this article in Portable Document Format (PDF) will represent a published work according to the International Commission on Zoological Nomenclature (ICZN), and hence the new names contained in the electronic version are effectively published under that Code from the electronic edition alone. This published work and the nomenclatural acts it contains have been registered in ZooBank, the online registration system for the ICZN. The ZooBank LSIDs (Life Science Identifiers) can be resolved and the associated information viewed through any standard web browser by appending the LSID to the prefix http://zoobank.org/. The LSID for this publication is: urn:lsid:zoobank.org:pub:4E0CF077-2DA5-451D-934E-103801608A36. The online version of this work is archived and available from the following digital repositories: PeerJ, PubMed Central SCIE and CLOCKSS.

### Taxonomy

**Table:** 

Pterosauria Kaup, 1834
Breviquartossa Unwin, 2003
Monofenestrata Lü et al., 2010
*Laueropterus* gen. nov.
*Laueropterus vitriolus* sp. nov.

*Diagnosis*

A moderate to large early pterodactyliform (non-pterodactyloid derived monofenestratan) that can be diagnosed by the following unique traits of this grade: a short wing phalanx 1 compared to 2 (80% of the length); wing phalanx 4 longer than wing phalanx 1 (also seen in *Darwinopterus robustodens*); humerus very straight with almost no curvature to the shaft (also seen in *Makrodactylus*); humerus shorter than the femur (also seen in *Skiphosoura*); greatly expanded proximal end of the wing metacarpal (large ventral expansion) that is double the width of the distal end; posterior ramus of the ilium long and with gentle ventral curvature.

*Holotype*

Specimen LF 6268 (Fig. 1) is a partial skeleton consisting of most of the cranium and a complete mandible, most of the cervical and dorsal vertebrae, the sacrum and some caudal vertebrae, pectoral and partial pelvic girdles, and most elements of all four limbs. The specimen is mostly disarticulated and largely compressed into two dimensions.

*Etymology*

*Laueropterus* meaning Lauer’s wing (pterus = wing in Ancient Greek), in honour of René and Bruce Lauer of the Lauer Foundation for Paleontology, Science and Education and the work they have done to make fossils, and especially Solnhofen region specimens, available for public study. Without their work, a number of important specimens might never have become available to science. The species epithet *vitriolus* from the Ancient Greek for ‘acid’ refers to the unusual pitting and holes seen the in the elements of the skeleton that look as if they have been dissolved away.

**Description**

The specimen LF 6268 is preserved on a thick slab of limestone that measures roughly 60 cm by 45 cm. The slab is mostly grey in colour but with thick white bars that run perpendicular to one another and cross in places (Fig. 1).

The pterosaur is generally very well preserved and undistorted, and thin elements such as the sternal plates clearly show the outlines of elements beneath them. Nevertheless, at least some elements are partially preserved in three dimensions. The specimen is largely disarticulated with major elements having separated from one another as is common for pterosaurs from the locality (*e.g.*, see Hone et al., 2023; Hone et al., 2024). The cranium has separated into several parts (although the mandible remains articulated), the radius and ulna have come apart, and the vertebrae are mostly scattered and separated, and not preserved as a series. However, even some very small elements are present (*e.g.*, caudal vertebrae) and some elements remain close to their natural articulation (*e.g.*, the wing finger), and there is no clear orientation of elements to suggest a current in any particular direction.

In terms of the ontogeny of the specimen, this shows a fully fused premaxilla-maxilla, but other major parts of the skull have separated out. Such separation appears to be common in the early monofenestratans, even large ones and otherwise apparently adult or near adult individuals like *Skiphosoura* (Hone et al., 2024), and so may not provide any indication of ontogeny (see Hone et al., 2025). Comparing the skeleton to the ontogenetic stages (OS) criteria of Kellner (2015) shows that *Laueropterus* has a late stage of maturity. There are no epiphysic plates or suture lines visible on any of the longbones which places this to at least stage OS2. The sacrum is incompletely fused as can be seen by the slight separation of some of the vertebrae which would indicate that it is between OS2 and 3, but the wrist elements are fully fused which is stage OS3 and above. The specimen also shows a fully fused scapulocoracoid with an obliterated suture, and the extensor tendon processes on the first wing phalanges are similarly fully fused, and the pelvic plates have separated from the sacrum placing this at OS 5–6. As such, despite the incompletely fused sacrum, this specimen shows enough features that, coupled with the size, means that osteologically it is mostly likely at adult or near-adult status.

The bone surface texture here is grainy which is usually a condition seen in only the youngest pterosaurs (Bennett, 1995) and there is a very fine texture of raised dots on flat areas (the premaxilla, sternum, prepubes, pelvic plates, neural spines). However, given that this animal cannot be that young given the extensive fusion, and the unusual taphonomy and preservation of the bones (see below), this texture is interpreted here as being a taphonomic artefact.

The specimen is clearly identifiable as a monofenestratan based on the large skull, confluent nasoantorbital fenestra, and the relatively elongate neck vertebrae (Lü et al., 2010). However, it also possesses a relatively short wing metacarpal and a short first wing phalanx that is not seen in the pterodactyloids, and so places this animal within the non-pterodactyloid grade (Lü et al., 2010).

The animal is moderate to large compared to other non-pterodactyloid monofenestratans. The total wingspan of the animal is c. one m (twice the total of one humerus, ulna, wing metacarpal and wing finger phalanges), which is only around half the size of *Skiphosoura* (Hone et al., 2024), but as large or larger than any of the other members of this grade that are known (see Hone et al., 2025). However, as it may have continued to grow further during its ontogeny, it may have been larger at full adult size (see Table 1 for a list of measurements).

*Skull*

Although the cranium has separated into a number of pieces, the major premaxilla-maxilla complex is complete as a single piece and is seen with the right side exposed, and the skull roof present as another seen in dorsal view, with some other parts also on the slab. The overall skull shape and size are therefore relatively clear. This would have had a typically monofenestratan triangular skull. The estimated length is 205 mm, long based on scaling the complete mandible preserved here against that of other related taxa (*e.g.*, *Makrodactylus*, (Hone et al., 2024); *Propterodactylus*, Spindler, 2024).

The suture between the premaxilla and maxilla is not visible. There is a slight line in the bone that may represent this, but it tracks very close to the dorsal margin of the premaxilla and would mean only one tooth would be in the maxilla and so this is considered most likely to be a taphonomic artefact. There is a small dorsal expansion to the anterior part of the premaxilla, that may represent a small bony crest (as in *Makrodactylus*, Hone et al., 2025), though one without the filigree margin as usually seen in other members of this grade (*e.g.*, *Darwinopterus*—Lü et al., 2011a; Lü et al., 2011b, *Skiphosoura*, Hone et al., 2024). The large and confluent nasoantorbital fenestra (NAOF) as seen is 110 mm long and 44 mm tall, though it lacks the elements that make up the posterior margin of this opening. The premaxilla-maxilla complex shows 11 teeth, though it is likely that one is hidden behind the wing element that overlies the tooth row. This is considerably more than the seven seen in *Makrodactylus* (Hone et al., 2025) and some darwinopterans (*e.g.*, *D. robustodens* with nine—Lü et al., 2011a; Lü et al., 2011b), though similar to the 11 of *Propterodactylus* (Spindler, 2024) and 12 of *Skiphosoura* (Hone et al., 2024), and less than the 15 of *D. modularis* (Lü et al., 2010) or 18 in *Darwinopterus camposi* (Cheng et al., 2025). Inside the NAOF, a long and thin piece of bone is visible that is a major part of the palatine, and alongside it is a very thin and rod-like element that represents parts of one of the vomers.

**Table 1:** Measurements of the major osteological elements. Measurements of the key elements of LF 6268.

**Element**	**Length (to the nearest mm)**
Skull	205 (estimated)
Mandible (midline)	158
Humerus	67 (L) 67 (R)
Radius/Ulna	105 (L) 103 (R)
Wing metacarpal	46 (L) 48 (R)
Wing phalanx 1	75 (L) 77 (R)
Wing phalanx 2	92 (L)
Wing phalanx 3	93 (L)
Wing phalanx 4	78 (L) 75 (R)
Femur	73 (L) 70 (R)
Tibia	97 (L) 96 (R)
Longest Metatarsal	27

A large piece of the skull roof that is 74 mm long sits next to the mandible (Fig. 2). This consists of the large and paired frontals, and posteriorly the paired parietals. These elements are all at least partially fused together as shown by their association, despite being separated from the rest of the cranium. The frontals are roughly triangular in shape and are similar in shape and proportion to that of *Makrodactylus* (Hone et al., 2025), although proximally they separate so that the premaxillae can sit between them. Laterally there is a semicircular excavation into the frontals that represents the dorsal margin of the orbit. On the right lateral face of the frontal, sits the semi-circular lateral extension of the prefrontal that would sit over the anterodorsal margin of the orbit, and the rest of the prefrontal is a long tapering triangular extension that reaches anteriorly. The left prefrontal has separated from the left frontal and sits anterior to the frontal pair and shows an additional short and triangular medial extension that would sit under the frontal when correctly articulated. Measuring across the excavation of the frontals and not including the prefrontal extensions, the anteroposterior diameter of the orbit would be approximately 24 mm. Posteriorly to the frontals, the paired parietals would be roughly square in shape but with a very large semi-circular excavation for the upper temporal fenestrae which would be 13 mm in diameter.

Adjacent to the large section of skull roof, and close to what would have originally been a natural position, is a block consisting of the jugal, quadratojugal and quadrate. Again, these were at least partially fused to one another given they are in a natural articulation despite separation from the other major skull parts. The jugal is a large and triradiate element with a long and slender (42 mm long, by 3 mm wide) anterior projection with a slightly forked tip to accept the posterior ramus of the maxilla. The second ramus extends anterodorsally and is thin (two mm), but its length cannot be determined as it is covered by the frontals. This ramus forms an angle with the third and final ramus that projects posteriordorsally, and this is relatively short and broad (20 mm by 4 mm) and tapers to a point. The angle between the latter two projections is rounded, but shows that the ventral margin of the orbit would have been somewhat pointed so that this opening would have been pyriform in shape, perhaps similar to that seen in *Propterodactylus* (Spindler, 2024), but less like that seen in *D. linglongtaensis* (Wang et al., 2010) or *D. robustodens* (Lü et al., 2011a; Lü et al., 2011b). The quadratojugal sits mostly behind the jugal, but with a dorsal ramus that is triangular in shape and overlies the posterior ramus of the jugal. The quadrate is a near rectangular element being 36 mm long and 4 mm wide, and is angled posteriodorsally such that with the jugal and quadratojugal it forms much of the margin of the lower temporal fenestra. From these elements, this opening would be long and thin and sit posterior to the orbit. A second complex of some of these elements is seen in posterior view, next to one femur and overlapped by a wing phalanx. This is the right jugal, quadratojugal and pterygoid, the latter of which consists of a long plate-like element, and the quadratojugal can be seen to be a large and bulky element.

Both postorbitals are preserved and have separated from the rest of the skull. The left is seen in lateral view and is lying on top of the right wing metacarpal and next to the left. Each postorbital is a small and tri-radiate element with one long process that would articulate with the jugal. The right postorbital is in medial view and lies between the right humerus and left femur. Two more small pieces of bone that are likely cranial elements are on the slab, but they are very thin, not well preserved and cannot be identified. One sits under the right postorbital, and the other is next to the atlas-axis.

**Figure 3:**
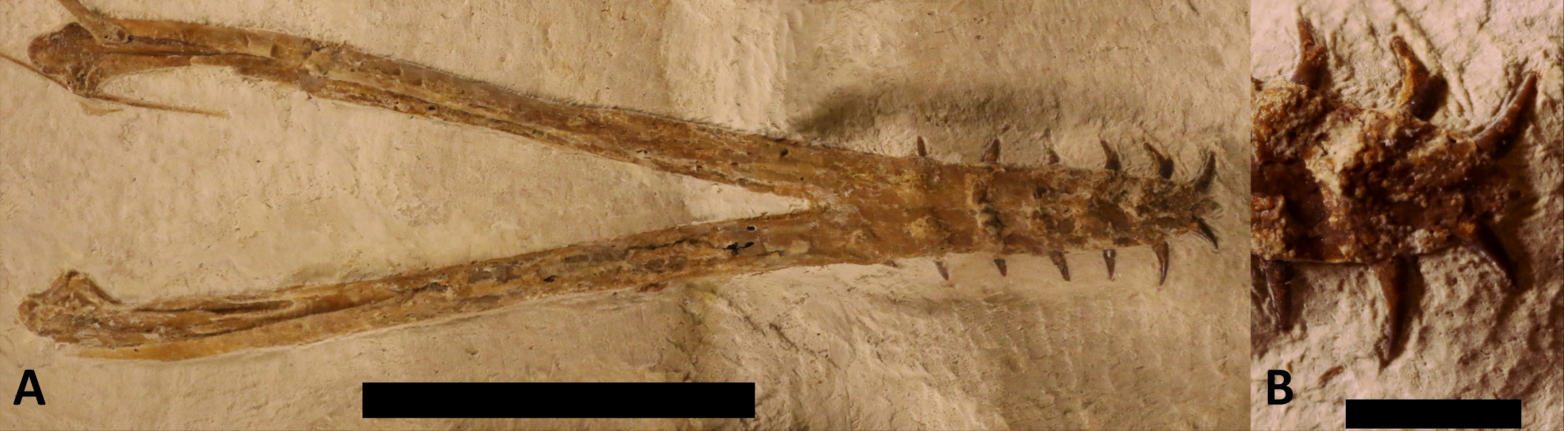
Mandible of LF 6268. Mandible as seen in ventral view. (A) General view of the robust element. Scale bar is 50 mm. (B) Close-up of the jaw tip, showing the orientation of the teeth and the bulges of the roots showing through the bone. Scale bar is 10 mm.

The mandible is preserved in ventral view and is complete and has not disarticulated (Fig. 3). This is 160 mm long (measured along the midline) and each ramus is 6 mm wide, and the maximum width of the jaw is 44 mm. This is similar to the proportions seen *Skiphosoura,* suggesting that they have similar jaw shapes, but *Makrodactylus* has a shorter and broader mandible, with a much longer mandibular symphysis (Hone et al., 2025–47% of the length, *vs* 35% here). The bulges of tooth bases show through the ventral face of the dentaries where these have been crushed during preservation. There are seven teeth in each dentary, and these begin close to the tip of the jaw and finish immediately anterior to the symphysis. The tooth count here is much lower than the 14 in *D. camposi* (Cheng et al., 2025), 12 illustrated for *D. modularis* (Lü et al., 2010, their figure 2c), the 11 seen in *D. robustodens* (Lü et al., 2011a; Lü et al., 2011b), but comparable to the seven or eight of *Skiphosoura* (Hone et al., 2024) and six in *Makrodactylus* (Hone et al., 2025) The mandibular symphysis is 53 mm long, making up about a third of the length of the jaw.

The semicircular articulars extend posteriorly from the mandible and these extend as tapering rods anteriorly, and are at least 49 mm long. Anteromedially to these are the thin and plate-like prearticulars.

*Dentition*

The teeth are generally long, thin and terminate in sharp points (Figs. 1–3) as in the other known Solnhofen-region early monofenestratans (Spindler, 2024; Hone et al., 2024; Hone et al., 2025). This is in contrast to a number of early monofenestratans where these are short and described as being spike-like or peg-like (Wang et al., 2010), and most notably in *D. robustodens* (Lü et al., 2011a; Lü et al., 2011b). The anterior teeth in *Laueropterus* are long and slightly curved and the posterior teeth (especially in the maxilla) are rather smaller and straight (Figs. 2 and 3). The longest tooth in both the upper and lower jaws is eight mm in length and the smallest c. four mm, and all are about two mm wide at the base. The very tip of the mandible is edentulous, unlike the right premaxilla which has a tooth present right at the very tip of the jaw. The first few teeth in the cranium are closer together than the others, but spacing of the teeth is generally consistent of 5–6 mm in the upper jaw and 5 mm in the bottom. The total tooth row of the upper jaws is 88 mm with the last three teeth positioned under the NAOF (as in *Skiphosoura*, Hone et al., 2024, but unlike *Makrodactylus,* Hone et al., 2025 where these terminate well before the NAOF) and in the lower jaw this is 46 mm finishing at the posterior end of the symphysis.

The teeth of the upper jaw are vertically orientated, but the orientation of the mandibular teeth shows them to be splayed laterally. This could be crushing given different orientations, but this is similar to the condition seen in *Makrodactylus* (Hone et al., 2025) and notably differs from that of *Skiphosoura* (Hone et al., 2024), both of which have the upper and lower jaws in the same orientations as here. This therefore suggests that the difference is genuine and not taphonomic in origin. As some additional support for this interpretation, the bases of the mandibular teeth in *Laueropterus* actually stick out through the ventral jaw in places (Fig. 3B), and are clearly close to the midline of the jaw, which matches only if they were already splayed naturally and were not sitting vertically in the jaw.

*Vertebral column*

**Figure 4:**
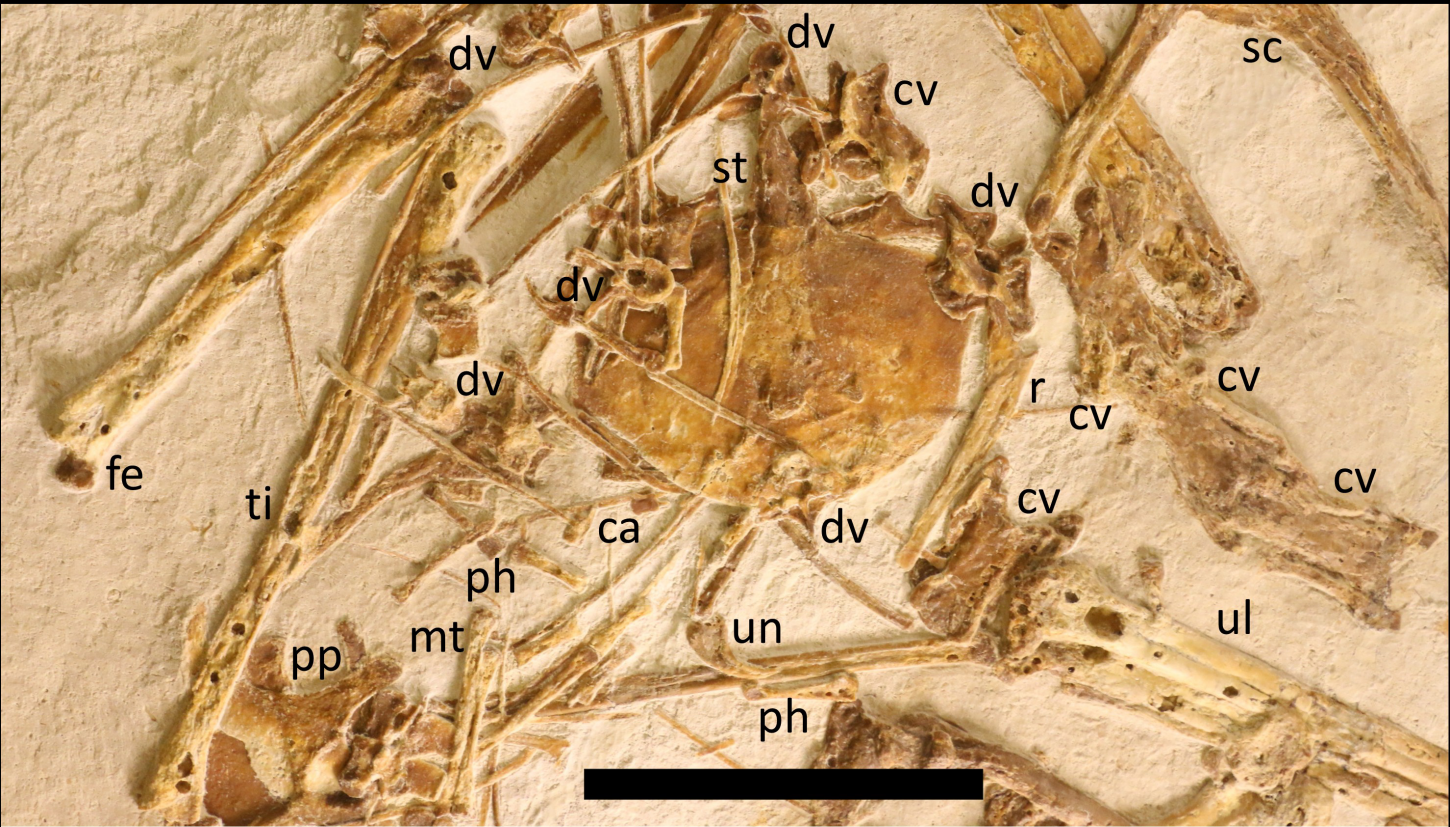
Major axial elements of LF6268. Postcranium shown includes partially articulated cervical vertebral series in dorsal view, dorsal vertebrae in anterior view, ribs, and the sternum in ventral view. Labels as in Fig. 1. Scale bar is 50 mm.

Most, if not all, of the vertebral column is preserved except part of the tail (Figs. 1 and 4). These elements vary in degrees of articulation and in orientation.

A total of nine cervical vertebrae are preserved, as is normal for non-pterodactyloids (Lü et al., 2010). There is an atlas-axis complex visible in anterior view. This shows two major depressions or holes, an upper oval one and a lower circular one. The upper is considered to be the neural canal and the lower a depression that contains some matrix that appears to penetrate the bone but does not, and so is likely an articular surface.

Next to the atlas-axis is an anterior cervical vertebra (Fig. 4), here considered to be CV 3 seen in anterodorsal view, and then next to this CV4 in dorsolateral view. Three cervicals are articulated in series nearby (CV 5-7?) and then one close to the sternum and radius and ulna (CV 8?) and a final one (CV 9?) that is close to the first dorsal vertebra, and all are seen in ventral view. These series positions are provisionally identified by their size, shape and position on the slab. None of the cervical series bear cervical ribs. C4 and C8 have centra that are approximately 18–20 mm long, and CV6 and 7 are the longest at 23 mm each. The centra of the cervical vertebrae are slightly constricted in the middle part and so are narrower here than at either end. In each, the prezygapophyeses are relatively short and do not extend far from the centrum either anteriorly or laterally, and the postzygapophyses are similarly short and close to the central body as also seen in *Skiphosoura* (Hone et al., 2024). The one visible neural spine (on CV3) is roughly triangular in shape and is 10 mm long and five mm high, and does not reach the anterior part of the centrum. These vertebrae are fundamentally similar to those in other darwinopterans (*e.g.*, Lü et al., 2010; Lü et al., 2011a; Lü et al., 2011b; Wang et al., 2010; Spindler, 2024) but these are generally not well-preserved, or seen in lateral view, and so are difficult to compare, except that of *Kunpengopterus* (Cheng et al., 2017) where the cervical vertebrae 3–5 are a very close match for those seen here in *Laueropterus*.

**Figure 5:**
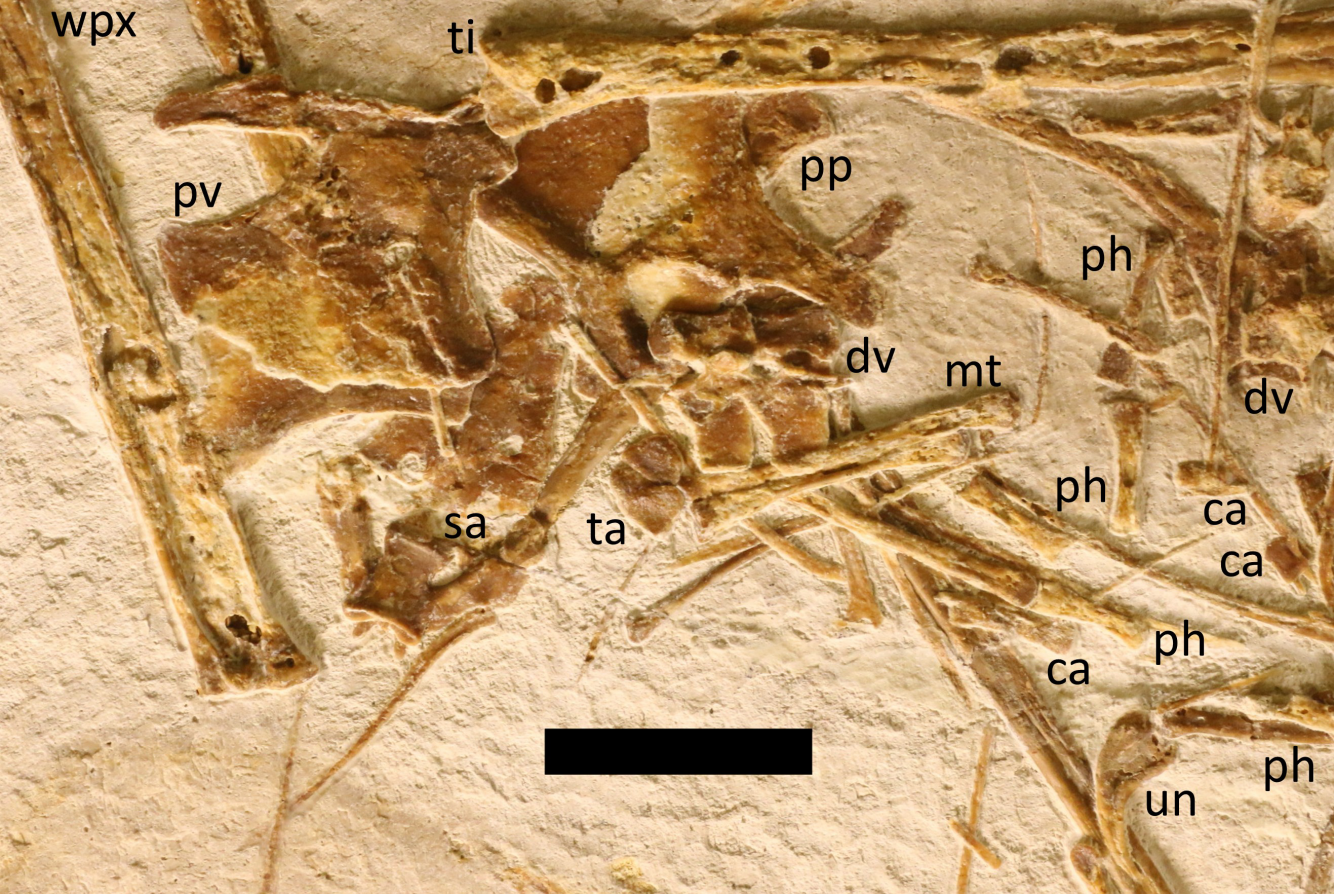
Close up of the pelvic girdle and associated elements of LF6268. Close up of the pelvic plates, sacrum and includes elements of the hindlimbs and caudal series. Labels as in Fig. 1. Scale bar is 20 mm.

The dorsal vertebrae are nearly all separated from one another (only two lie in articulation, and these are next to the sacrum—Figs. 4 and 5). A total of 14 dorsals are preserved here and this may be the complete series. Lü et al. (2010) noted that non-pterodactyloids typically have 26 pre-sacral vertebrae, with nine in the cervical series. However, *D. robustodens* is preserved complete and in articulation and is described as having only 21 (eight cervical and 13 dorsal Lü et al., 2011a; Lü et al., 2011b) and so this lower value is also potentially present in at least some other early monofenestratans. The dorsals are seen mostly in posterior and lateral views, with two in anterodorsal view. The first dorsal vertebra is identifiable as it is both large and with a large neural arch, and the very large and broad anteriormost dorsal ribs are still in articulation with it. This is the largest dorsal, though it is crushed in anterodorsal view making the details difficult to determine or the exact shape of the bone. One relatively proximal dorsal (based on its size) seen in dorsal view shows very wide prezygapophyses (13 mm apart) which is the same as the length of the centrum in this element, and much wider than the postzygapophyses here (8 mm apart). These are fundamentally similar to those of *Skiphosoura* (Hone et al., 2024), though here the lateral processes are less robust.

Several smaller and more posterior dorsal vertebrae show the details of these elements more clearly (Fig. 5). These have centra that are seven mm long and five mm in diameter and have a straight ventral margin. Anteriorly, the centrum is hemispherical, and the posterior face is oval shaped to U-shaped with a depression in the upper part for the circular neural canal. The lateral processes extend up to six mm from the centra, and are angled slightly dorsally. These are fundamentally similar in shape to those seen in *Ceoptera* (Martin-Silverstone et al., 2024) and *Skiphosoura* (Hone et al., 2024). The neural spine is slightly smaller being up to five mm tall and six mm wide, it is rectangular in lateral view. These lack the slight posterodorsal rounded expansion on the neural spines that is apparently unique to *Propterodactylus* (Spindler, 2024). In total there are 14 dorsals present including two that are preserved under the sternum (but their outlines are clearly visible) and a sacralised dorsal that sits with the sacral series.

A sacral series is preserved in ventral view and consists of four rapidly tapering centra with three sacral ribs (Fig. 5), and the last having only the start of these processes, but they would not be long enough to reach the pelvic plates and have not joined the preceding ribs. The shape and structure of this is similar to that of other pterosaurs and notably close to that seen in the well-preserved sacrum of *Kunpengopterus* (Cheng et al., 2017). The sutures between the sacral vertebrae are incompletely obliterated. The sacralised dorsal that sits with the sacrum to form a synsacrum appears to be a separate element, but this is as a result of some damage to the point where it contacts the first sacral vertebrae and it is likely that this was at least partially fused to the sacral block.

The caudal series is very incomplete, but there are four caudal vertebrae preserved on the slab close to the sacrum (Fig. 5), and probably a fifth below the sternal plate. These are all similarly sized, being around four mm long and two mm wide, with the centra having circular faces and being slightly constricted in the middle part. The prezygapophyses are small and pointed and extend only a millimeter or so from the centra (Fig. 5). The vertebrae are small elements, but are not especially short as would be expected for the most anterior caudals and they would be too small for this position based on the size of the last sacral centrum. These are therefore considered mid-caudals since the distalmost ones would be very short and simple. There is no indication of elongate zygapophyses or chevrons which are seen in most non-pterodactyloids monofenestratans (Hone et al., 2024). The length of the tail cannot be estimated from this limited data, but this would imply that the tail would be short overall based on the lack of supporting rods seen in *Skiphosoura* and earlier branching members of the grade (Hone et al., 2024), but that are greatly reduced in the derived *Propterodactylus* (Spindler, 2024).

Various dorsal ribs and gastralia are scattered across the slab, and only the large proximal pair of dorsal ribs are articulated with a dorsal vertebra (Figs. 4 and 5). The disarticulated elements vary in thickness and curvature and some elements are very thin. The ribs are typically 40 mm long and around 2 mm wide, while the gastral elements may be a similar length but half the diameter or less. Several of the latter are distinctly V-shaped, as is normal for pterosaur gastralia.

*Girdles and limbs*

**Figure 6:**
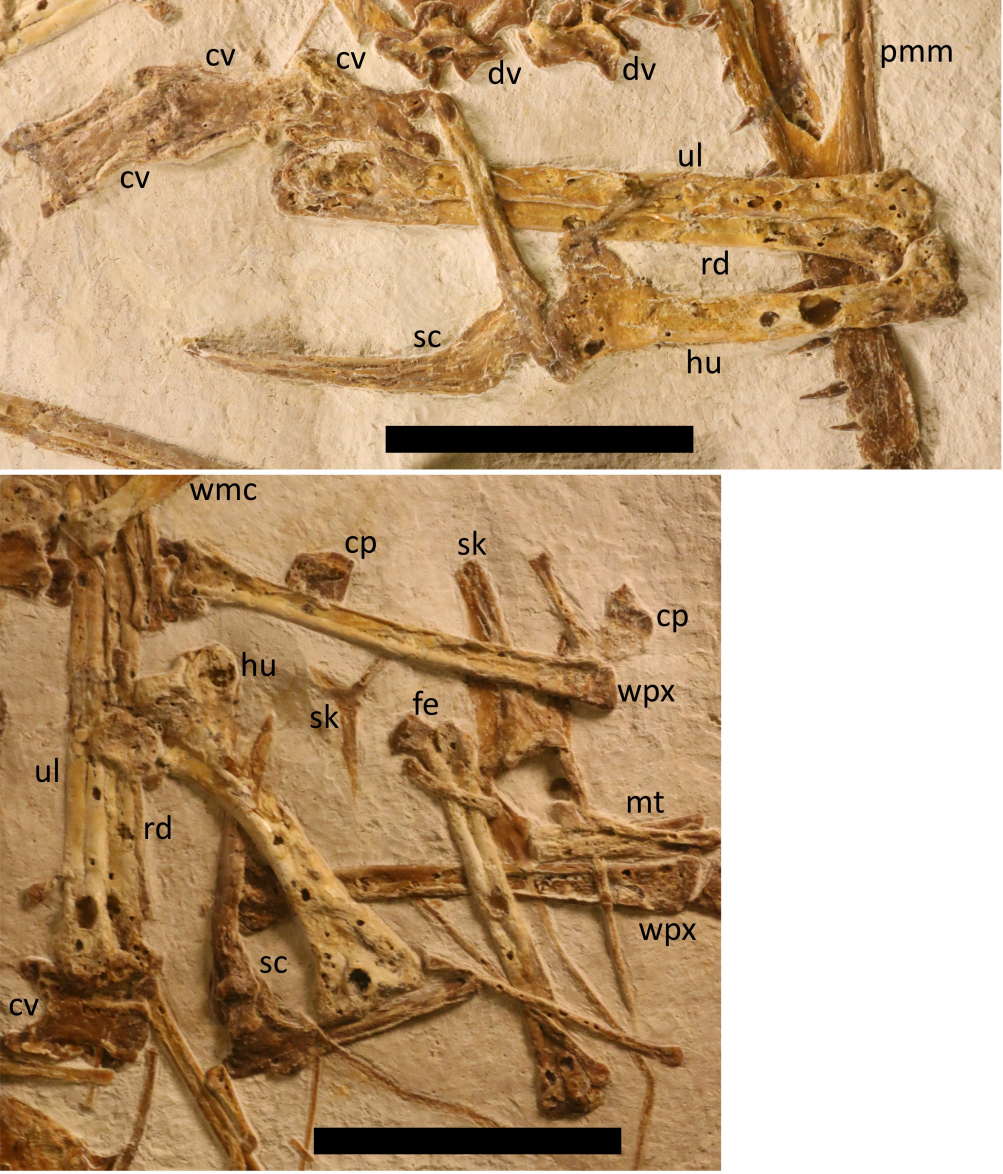
Pectoral girdle and forelimbs of LF6268. Pectoral girdles and wings of the holotype. (A) left wing (B) right wing. Labels as in Fig. 1. rd, radius. Scale bars are 50 mm.

Both scapulocoracoid pairs are preserved and are fully fused (Figs. 1 and 6), the left is seen in lateral view with clear glenoid, and the right is in medial view. The scapula is the longer element (54 mm to 47 mm) with the coracoid having a slight lateral extension that protrudes beyond the glenoid, and the shafts are a similar thickness (four mm). The scapular appears to lack the expansion at the end of the dorsal ramus that is seen in other taxa (see Martin-Silverstone et al., 2024, including *Skiphosoura*—Hone et al., 2024), though this may be hidden in the matrix or worn. The shaft of the scapula is much narrower than seen in *Makrodactylus* (Hone et al., 2024). The glenoid is saddle-shaped with dorsal and ventral expansions, and based on its elevated position, sits primarily on the scapula. It bears a strongly projecting biceps tubercle (acrocoracoid) as seen in other early monofenestratans, (Martin-Silverstone et al., 2024), but is notably larger than that seen in *Skiphosoura* (Hone et al., 2024) and especially *Makrodactylus* (Hone et al., 2025).

A well-preserved and complete sternum is present in ventral view (Fig. 4). The cristospine is short (13 mm long) and robust with slight lateral expansions where it meets the sternal plate, and a ridge along the top of the cristospine appears to extend a short way onto the face of the plate. The sternal plate is deep and broad (37 mm by 51 mm wide) has slightly posteriorly directed anterior margins, straight sides and then a gently rounded posterior margin. The plate is clearly very thin given the details visible of elements preserved below it, but it appears to be well ossified and fully fused to the cristospine. The shape of this is more rounded than is often seen in other darwinopterans (*e.g.*, Hone, 2023; *Makrodactylus*, (Hone et al., 2025), and is closer to the outline seen in *Skiphosoura* (Hone et al., 2024–although this is not well-preserved).

The two prepubes are present, and these are close to one another and the pelvic plates, but one is largely covered by a plate and the other is poorly preserved with the anterior margin being largely missing (Fig. 5). These can, however, be seen to be typical of monofenestratan prepubes (*e.g.*, see Lü et al., 2011a; Lü et al., 2011b; Spindler, 2024; Hone et al., 2025) with a somewhat semicircular main part that is 23 mm across, and then a rectangular posterior expansion that here is c. 10 mm long.

The two pelvic plates are similarly hard to make out, but the left one, seen here in medial view, is clearer (Fig. 5). It shows complete fusion of the ilium, pubis and ischium into a single piece, which is a typical shape for pterosaurs, and bears a small (two mm) circular opening close to the anterior face. The anterior ramus of the ilium is hidden below the tibia, but the posterior rami of the ischia are visible on both plates, and these are long and have a slight ventral curve along their length. A similar curvature is seen in *Ceoptera* (Martin-Silverstone et al., 2024) although this is a much shorter and deeper ramus. The plate has a slightly concave anterior margin and a more sharply curved concave posterior margin and a gently convex ventral margin, but lacks the hook-like anterior expansion seen in *e.g.*, *Kunpengopterus* (Cheng et al., 2017), or the straight ventral margin in *D. robustodens* (Lü et al., 2011a; Lü et al., 2011b) and *D. linglongtaensis* (Wang et al., 2010). Across the ventral face, the plate is a maximum of 39 mm wide. Although partially covered, the right does have the acetabulum exposed and this is circular with a diameter of five mm.

Nearly all parts of both wings are preserved (Fig. 1) with only one pteroid and possibly some carpals or elements of the free fingers missing or hidden on the slab. These vary in articulation with, for example, the right scapulocoracoid, humerus, radius and ulna are articulated, and one wing finger being in near natural articulation, though the other is separated and somewhat scattered.

The two humeri are preserved (Fig. 6) and both appear to be unusual in having a very straight shaft. This is considered to be genuine and not a result of their orientation as the two lie in slightly different planes, and all other early mononfenestratan humeri are curved to at least some degree (see *e.g.*, Wang et al., 2010; Lü et al., 2010; Lü et al., 2011a; Lü et al., 2011b; Cheng et al., 2017; Spindler, 2024). This curvature is very minor in *Douzhanopterus* (Wang et al., 2017) but still present, and in this taxon, the humerus is both proportionally thinner and the deltopectoral crest is more triangular and less rounded than seen here. Both humeri in *Laueropterus* are seen in largely lateral view, though the left humerus is somewhat rotated such that the deltopectoral crest is partly broken, the medial crest is visible, and the distal end is seen in anterior view. The deltopectoral crest is a broad and slightly triangular expansion that has a rounded distal margin and descends to a quarter of the way down the humeral shaft. The medial crest is slightly smaller and more rounded. The lateral condyle of the distal part of the humerus is much larger (more laterally developed) than in other animals of this grade. Overall, the humerus is very similar to that seen in other early monofenestratans including *D. modularis* (Lü et al., 2010, their fig 2f), *K. sinensis* (Cheng et al., 2017) and *Propterodactylus* (Spindler, 2024), thought he deltopectoral crest is smaller than seen in *Skiphosaoura* (Hone et al., 2024).

The left radius and ulna are preserved in articulation (Fig. 6A), with the wing metacarpal next to them, and this is in articulation with the left first wing phalanx, but only the latter element is not covered by other bones. The right radius and ulna (Fig. 6B) are also preserved together and these are more clear. They are long, and straight and simple elements that are subequal in length and diameter (103 mm by 6 mm) as is normal for early monofenestratans.

Three syncarpals are visible on the slab, one lies next to the left first wing phalanx, a second near the end of this element and a third by the distal end of the right wing metacarpal (Fig. 6B). As such, none are close to their original articulations and have all moved from their original positions, despite the general articulation of the wings seen here. All three syncarpals are blocky and roughly square elements that are around 10 mm in diameter, and their details are difficult to make out. One pteroid is preserved, and this is curved along its length, relatively short (38 mm long, measured in a straight line) and thin (two mm midshaft diameter) and has a rounded proximal end. This is more curved than seen in many early monofenestratans *e.g.*, *D. robustodens* (Lü et al., 2011a; Lü et al., 2011b), *Douzhanopterus* (Wang et al., 2017), and *Propterodactylus* (Spindler, 2024), but is similar in curvature and robustness to that of *Skiphosoura* (Hone et al., 2024).

The right wing metacarpal is preserved in anterior view (Fig. 1). The proximal end is unusually robust, being nearly double the width of the distal condyle (14 mm *vs* 8 mm) much more than seen in *Ceoptera* (Martin-Silverstone et al., 2024) or *Skiphosoura* (Hone et al., 2024), though similar to that seen in *Makrodactylus* (Hone et al., 2025). Metacarpals 1–3 have separated from the wing metacarpal and are mostly missing, but one can be identified. It is the same length as the wing metacarpal but is only two mm in diameter and has a slightly expanded and squared off proximal end, and a hemispherical distal end.

All of the wing phalanges are preserved on the slab (Fig. 1), but one wing (probably the right) is entirely separate and the other articulated. All of these are typical for pterosaurs being generally long and slender elements. The first wing phalanges have fully fused extensor tendon processes, and the fourth wing phalanges taper to a point and are slightly curved along their length. The series is unusual in that wing phalanges 2 and 3 are near identical in length, with 4 and 1 being a similar length, though 4 is the longer of these two. This pattern is not normally seen in darwinopterans or other pterosaurs, though it is close to the unusual condition in *Makrodactylus* where wing phalanges 1–3 are all similar lengths (Hone et al., 2025).

Both femora and tibia are present and seen in anterior view, with the right tibia in near articulation with the right femur (though at an unnatural angle) and the left tibia separated but close to the left femur (Figs. 4 and 6). The femoral head is offset at an angle of 50 degrees from the shaft and has a thin neck with a small hemispherical head, and the left shows that the fourth trochanter is quite large and extends c. four millimeters down the shaft. The shaft of the femora is straight and terminates with a moderate expansion and into a pair of condyles. This femur is robust as in *Skiphosoura* (Hone et al., 2024) and *Propterodactylus* (Spindler, 2024) and much thicker than seen in *e.g.*, *Coeoptera* (Martin-Silverstone et al., 2024) or *Douzhanopterus* (Wang et al., 2017).

The tibiae and straight and slender, though the midshaft is slightly narrower (five mm) than the proximal and distal parts of the shaft (seven mm) that precede the condylar expansions. On the right tibia, the fibula is also visible. The proximal end of this is hidden by a gastralia, but the shaft of the fibula is very thin (one mm) and extends little more than a third of the way down the tibia before fusing into it, being only 33 mm in length. The tibia is long compared to the femur (ratio of femur/tibia of 0.75) compared to other local monofenestratans (ratio of 0.82 to 0.86—see Hone et al., 2025) and is much less than *Douzhanopterus* (0.57—Wang et al., 2017), but is similar to *D. modularis* (0.77—Lü et al., 2010) and *K. antipollicatus* (0.74—Zhou et al., 2021).

One set of three distal tarsals are present on the slab, adjacent to a set of metatarsals but separate from the distal right tibia (Fig. 5). These are small elements, two of which are semicircular elements that are five mm in diameter and sit next to one another and a third that appears to be slightly smaller and circular and is preserved beneath them. The distal tarsals sit adjacent to a cluster of three closely appressed metatarsals, two of which are clearly visible and a third sits behind these two. What is likely the fourth metatarsal, protrudes at an angle from these and is articulated with a pedal phalanx. A second set of metatarsals lies close to the left femur, these are also closely appressed but lie on top of one another so that only three are clearly visible. All of these elements are long, thin and straight which are subequal in length being circa 27 mm and 2 mm in diameter.

Various manual and pedal elements are scattered across the slab and very few are in articulation or even close to other elements (Fig. 1). As a result, it is not possible to easily restore the hands and feet, but they are clearly similar to those of other pterosaurs with the manual elements being larger and more robust than the pedal ones. Unguals for both the hands and feet are preserved, and these have similar shapes to one another. In both cases these are relatively long and strongly curved and sharp. Such a pattern is seen in some other early monofenestratans, *e.g.*, *Kunpengopterus* (Cheng et al., 2017; Zhou et al., 2021) and *Skiphosoura* (Hone et al., 2024), but in *Propterodactylus* for example, the pedal unguals are rather shorter and less curved than those of the manus (Spindler, 2024).

**Discussion:**

*Identity*

*Laueropterus* is clearly an early monofenestratan pterosaur, combining the pterodactyloid trait of a large head with confluent nasoantorbital fenestra with the non-pterodactyloid traits of a long wing metacarpal and wing finger proportions where phalanges 2 and 3 are longer than 1. Moreover, it is taxon that is likely close to the recently named *Skiphosoura* (Hone et al., 2024) as it is also a large monofenestratan with similarly large and fang-like teeth that are well spaced, a generally similar head shape, a robust mandible, robust but strongly curved pteroid (though it is proportionally shorter here), robust femur, and what was likely a short tail based on the preserved caudals. Despite these gross similarities (and some details, such as the humerus being shorter than the femur and the relatively long ulna—see Table 2), there are also a number of clear anatomical differences between the two (in addition to the unique features given in the diagnosis). *Laueropterus* is not as big an animal (though could have grown rather larger) and in the skull, bears a smaller crest on the premaxilla which lacks the filigree texture of *Skiphosoura* (though this could be ontogenetic or dimorphic), the snout is longer and less tall here (total length of the premaxilla and maxilla *vs* the maximum height of the latter 2.93 *vs* 3.27) and the lower jaw here is longer and thinner (c. 3.7 times longer than wide, *vs* c. 3.2). In the postcranium, the dorsal and lateral margins of the sternum make this more shovel-shaped than the fan-shape of *Skiphosoura*, the lateral condyle of the distal part of the humerus is much larger here, notably also wing phalanx 4 is longer than 1, and the femur not as long compared to the tibia (0.83 *vs* 0.75), and there is the apparent absence of elongate binding rods on the caudals in *Laueropterus*.

Other differences are also seen between *Laueropterus* and other early monofenestratans from the Solnhofen region. For example, although the ratio of the length of the humerus to the radius here is only slightly less than *Skiphosoura* (0.65 *vs* 0.67) it is much lower than the others (*Propterodactylus*, 0.72, *Makrodactylus* and “Rhampodactylus” are 0.76). Similarly, the pteroid is proportionally long here, with the humerus:pteroid ratio being 1.7, with the others being 1.9–2.2, and, as noted above, the femur is only 0.75 of the length of the tibia (data from Hone et al., 2025).

Although pterosaurs are not truly isometric (*e.g.*, see Yang et al., 2023), a high degree of isometry is seen (*e.g.*, see Hone et al., 2021) and intraspecific variation of features can also be low (*e.g.*, Bennett, 1996; Habib & Hone, 2024). Therefore, these differences are likely taxonomic and not the result of intraspecific variation or growth, and therefore give confidence that these proportional differences represent distinctive taxonomic traits. This similarly holds for other Solnhofen region early monofenestratans that also differ in their proportions to *Laueropterus* (see Table 2).

**Table 2:** Proportions of major elements of Solnhofen region early monofenestratans. This shows the distinctive ratios of many of these elements for all the taxa in addition to other morphological traits that help separate them out as distinct. The proximal wing is taken as the lengths of the humerus, ulna and wing metacarpal, with the distal wing being the combined lengths of the wing phalanges for one wing.

**Taxon**	**Humerus/ Radius**	**Humerus/ Wing metacarpal**	**Humerus/ pteroid**	**Wing phalanx 1/2**	**Humerus/ femur**	**Femur/ tibia**	**Proximal *vs* distal wing**	**Source**
*Laueropterus*	0.65	0.72	1.76	0.82	0.92	0.76	0.64	This study
*Makrodactylus*	0.76	0.57	?	0.92	1.10	0.83	0.62	Hone et al., 2025
*Propterodactylus*	0.72	0.64	2.12	0.91	1.03	0.87	0.66	Spindler, 2024
*‘Rhamphodactylus’*	0.76	0.73	2.15	0.94	1.08	0.82	0.77	Rauhut, 2012
*Skiphosoura*	0.67	0.71	1.91	0.86	0.91	0.83	0.69	Hone et al., 2024

*Taphonomy*

Most Mühlheim locality pterosaurs from the Mörnsheim Formation show a preservation pattern that is different to others of the Solnhofen region, with the specimens being partially or largely disarticulated, but largely complete and with 3D preservation (*e.g.*, Wellnhofer, 1975; Rauhut, 2012; Hone et al., 2023; Hone et al., 2024; Hone et al., 2025), which is similar to at least some other vertebrate specimens from the Formation (*e.g.*, fish Wilkin, 2019). Solnhofen region specimens are often complete and articulated, but where elements or skeletal units have separated, they are often lost (Beardmore, Lawlor & Hone, 2017) and they are generally compressed into two dimensions, and soft tissue is relatively commonly preserved (Frey et al., 2003).

In the case of LF 6268, this is more articulated than some other pterosaurs from the formation (there is a string of cervicals, the dorsals are in articulation, there is an articulated series of the scapulocoracoid, humerus, radius and ulna, wing metacarpal and one wing finger), but still a lot of separation (the skull roof, the pelvic plates) and a number of smaller elements such as the ribs, free fingers and toes are scattered or missing. As with other Mörnsheim pterosaurs (*e.g.*, Hone et al., 2023; Hone et al., 2024; Hone et al., 2025), there is no clear evidence of a palaeocurrent that would have aligned the smaller and long elements such as the ribs and wing finger phalanges and suggests that specimen hit the bottom of the lagoon largely or totally intact and was buried later.

**Figure 7:**
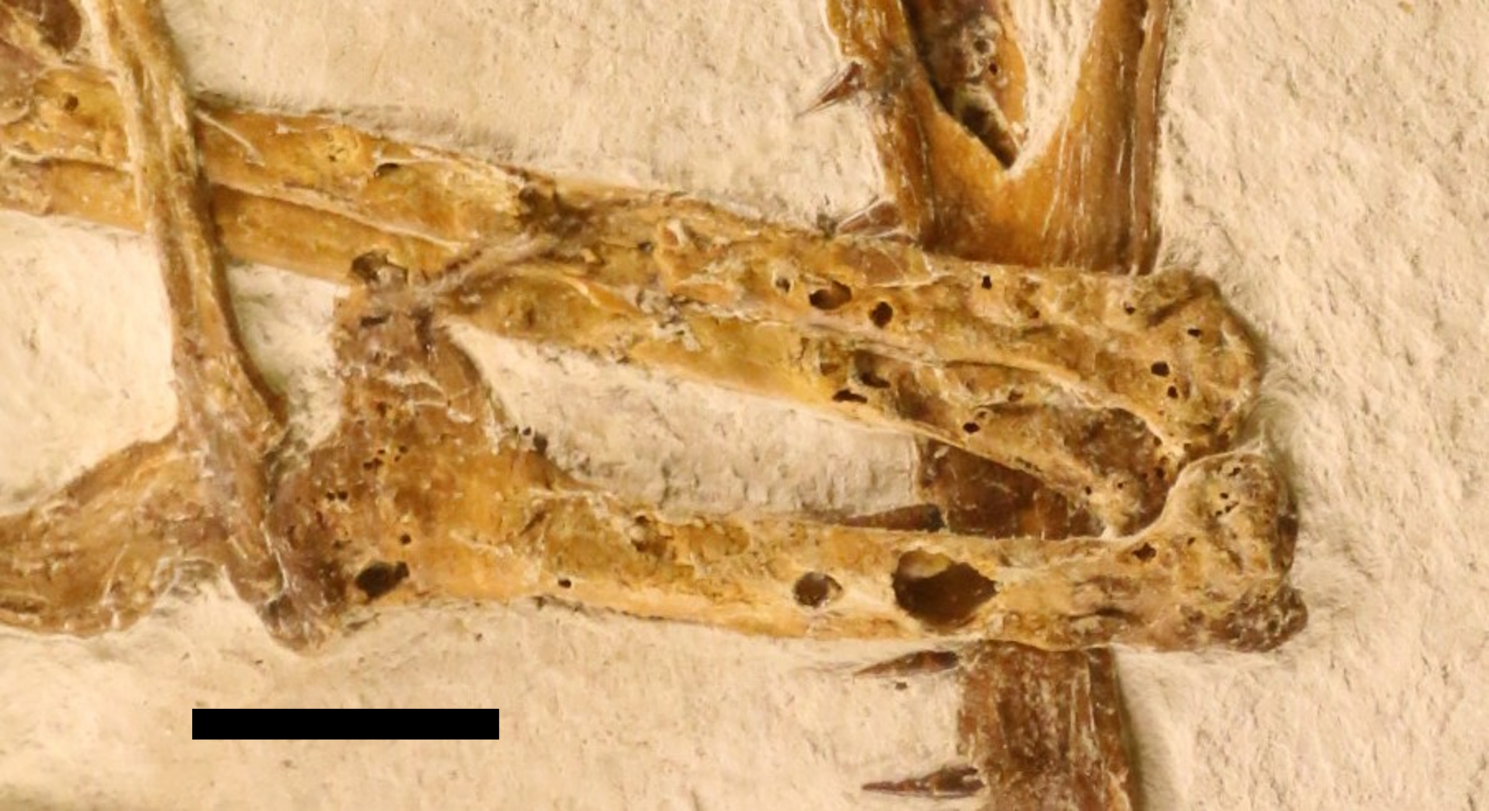
Close up of damage to the humerus and other elements. The image shows where the iron has leeched away, and holes have formed in the otherwise well-preserved elements. Contrast this to the sternum and pelvis in Figs. 4 and 5. Scale bar is 20 mm.

The holotype of *Laueropterus* also has some unusual, potentially unique, features in its preservation. Most notable is the orange tint to many of the bones (when initially studied, this was given the nickname of ‘redwing’), as well as the extensive pitting these have suffered (Fig. 7). An orange halo around bones is uncommon, but does appear in some Solnhofen pterosaurs specimens (*e.g.*, see Hone, 2012) but not the bones themselves, or at least not to this degree (*e.g.*, a specimen of *Pterodactylus*, BSPG 1969 I 91 is rather pink in colour). There is also the white ‘cross’ in the matrix that notably does not appear to have affected the bones much if at all.

The regular pattern of the paler stripes on the matrix are similar to those seen formed by conjugate sets (Sibson, 2000: see their figure 7) where cracks formed post deposition have been invaded by other material. This could be groundwater that has welled up or from rainwater than has seeped down from above alongside precipitation of different compounds has occurred along these cracks. The material can spread out along the original cracks altering the composition of the local rocks and so leading to thick bands of alternate colours, here the paler cross. Here the grey colour of the main matrix is considered to be rather silicified, and the white is more carbonate-based as this can be carried in water.

This pattern of water action is likely linked to both the bone pitting and the colouration of the bones. The pitting may be the result of dissolution of the bones (*e.g.*, see Hedges, 2002): specifically, this is likely to be calcium given the carbonates seen in the structures above that were likely in the water. With water contacting bones at the interface with the rock, the permeability of bones would leave them vulnerable to some parts being dissolved, leaving these pits. Although numerous elements show pitting, this is generally on the large elements (humerus, radius and ulna, femur, tibia, proximal wing phalanges) that also carry the least staining, whereas the smaller and flatter pieces (skull roof, sternum, pelvic plates, small ribs) have less pitting but are more orange.

In the partial 3D preservation of the specimen, larger elements would be taller and so water seeping down from above would contact these first. Therefore, they have tended to suffer more damage, while the smaller and thinner elements that are lower down were less affected. The orange tones may be a result of iron oxide deposits. In the absence of any staining of the rocks or any evidence of orange material percolating in the sediments, the obvious source here is the skeleton itself with blood providing a source of iron, though iron also appears to be a key component of the preservation of Solnhofen insects as well (Storari et al., 2024), so this may be a natural source from the rocks. A specimen of *Sinopterus* from the Lower Cretaceous Jiufotang Formation in China was described has having iron concentrations around the soft tissues, with iron negatively correlating with calcium (Lu et al., 2025), a contrast here where it is present in the bones, but not the surrounding matrix. Iron oxide formation in the holotype of *Laueropterus* would colour the bones but also protect them (this is reminiscent of what is seen in some trilobite fossils—Skinner, 2005) and so the pale bones are those where water has had the most contact, and then taken away the iron oxide as a protective layer and been able to dissolve the bones underneath.

The Mühlheim locality is also notable for the absence of small and/or younger pterosaur specimens (at least to date). These usually dominate in the Solnhofen region beds (Bennett, 1995; Bennett, 1996) in contrast to the pattern seen for Mesozoic dinosaurs which is dominated by larger animals and adults (*e.g.*, Hone & Rauhut, 2010). Young pterosaurs could fly (Bennett, 1995; Hone et al., 2021; Naish, Witton & Martin-Silverstone, 2021) and so if out over water were at risk of drowning and later burial. Even if they could take off from the surface (Pittman et al., 2022), pterosaurs as a whole might be vulnerable on water, which would exaggerate their presence in the fossil record (Hone & Henderson, 2014). Although pterosaurs were highly pneumatic and potentially able to float for extended periods before sinking (and this is supported by disarticulation patterns—Beardmore, Lawlor & Hone, 2017), any that died in open water could sink and be buried in anoxic conditions in the Solnhofen lagoons while on land they might be more likely to have encountered scavenging vertebrates, insects or decay on the surface or in the soil. Therefore, small (and usually young) animals which would be more numerous than adults tend to be destroyed on land, but can be preserved in water.

Although the numbers of pterosaur specimens are currently low from Mühlheim, this is not the pattern seen here. *Petrodactyle* and *Skiphosoura* are two of the largest pterosaurs in the region and *Laueropterus* (one m wingspan) and even *Makrodactylus* (c. 60 cm) are well over the size most pterosaurs seen in other Solnhofen region beds (see Wellnhofer, 1970; Wellnhofer, 1975). Animals with a skull length of 105 mm, humerus length of 42 mm or more (*i.e.,* larger than *Makrodactylus*) make less than one fifth of specimens in other beds (based on Bennett, 1996—his figure 1), but here this is the smallest known specimen. Despite the limited data so far, this suggests that one or more biases are in operation here.

To my knowledge, there has been no bias in the quarrying and excavation of smaller specimens and although smaller specimens will be harder to find, this clearly has not been an issue for finding them in other quarries in the region. Therefore, there is either some form of bias against preserving smaller specimens here, or they were rare, or not living in the area (or flying across it) and so were not captured.

It is possible that early monofenestratan pterosaurs had a different population structure to others with far fewer juveniles and more adults, but this does seem unlikely. This is not the pattern seen with either the preceding non-pterodactyloids (Bennett, 1995), or the early pterodactyloids (Bennett, 1996), and there are a number of young darwinopterans preserved in other pterosaur communities such as the Daohugou beds (Sullivan et al., 2014). The sole specimen of *Propterodactylus* is a young animal (Spindler, 2024) and is by far the smallest darwinopteran known from Germany and is from Painten, fitting the normal pattern of small specimens being prominent in these other localities (if as a single datapoint). As such, this hypothesis is not considered further.

It may be that smaller pterosaur specimens are not being preserved in the formation. To date, there have been limited reports of smaller vertebrate specimens (*e.g.*, some fish have been described—Wilkin, 2019), and none of the pterosaurs have shown any preservation of soft tissues which are again relatively common elsewhere in the region (*e.g.*, see Frey et al., 2003). So, there may be some form of systematic bias against more fragile specimens which would generally include younger pterosaurs which possessed both smaller and less well-ossified skeletons. As noted above, there appears to be no currents that would cause the loss of elements or change their orientation in the pterosaurs, but perhaps this is an issue only at depth. A light current in the upper waters could transport smaller bodies further and means they were brought past this system and so were not there in the first place, but larger ones moving more slowly were captured. Currents have been noted with the disarticulation of some fish specimens (Wilkin, 2019) though these elements are typically much smaller and easier to move. The relatively coarse preservation (as seen from the lack of soft tissues) would then put a further bias against small pterosaurs. Even if this was the case, this would still make the number of large specimens unusually common but would potentially explain at least some of the apparent absences of smaller specimens. We have now found about perhaps half the one meter plus wingspan pterosaurs in the region in a few years compared to centuries of excavations elsewhere in the region, so these are unusually common here, in addition to small things being rare.

Finally, this could be a fundamentally different locality in terms of the species and individuals that were locally present. Clearly there is a bias here towards this grade of animals with them making up the majority of the remains so far. These are considered to be better adapted to terrestrial environments (Smyth et al., 2024), and are generally common in terrestrial settings (Sullivan et al., 2014) and have been found alongside numerous anurognathids which are similar considered inland, terrestrial taxa (see Hone, 2020). In contrast, *Rhamphorhynchus,* which are by far the most numerous Solnhofen region non-pterodactyloids, are primarily considered marine animals (Bennett, 1995), and the numerous euctenochasmatoids were primarily piscivorous or filter feeders (*e.g.*, Witton, 2013; Witton, 2018) and so must have frequented, if not habitually occupied, coastlines and aquatic systems. Yet to date there is only one *Scaphognathus* specimen said to be from the formation (see Table 2), another member of lineage that is often considered to be more terrestrial in its habits.

Arratia et al. (2015) reported undescribed and privately held specimens of *Rhamphrohynchus*, *Pterodactylus*, *Ctenochasma* and the then unnamed *Petrodactyle* as being from the ‘Morosheim’ [sic]. However, the alleged *Pterodactylus* specimen is the ‘Rhamphodactylus’ specimen, and no specification of the locality was given for any of these, so they could potentially be from Mühlheim, Daiting or a mixture. The *Rhamphorhynchus* at least is likely from Mühlheim (B. Lauer, pers. comm., August 2025) but this would still leave this as a rare component of the fauna rather than being dominant as it is elsewhere in the Solnhofen. This implies that that the pterosaurs with more aquatic habits might not have been common in the area that produced the Mörnsheim fauna, as the pterosaurs we are finding are primarily inland species and not those that frequented large bodies of water.

There has so far been little comment on the fauna of the Mörnsheim to date. Foth et al. (2025) note that there are various undescribed reptiles including turtles, rhynchocephalians and crocodyliforms and that there are a large number of ammonites in the beds, and the presence of other invertebrates. Wilkin (2019) discussed some of the fish fauna, and suggested that the basin in which these finds have been preserved, is not capturing local habitual taxa, but instead the fish at least, are allochthonous and were brought in from reefs, given that the common fish are of weak swimmers and that benthic taxa are rare. Wilkin also noted that the fish did not show evidence of death through toxicity, suggesting that the waters were not toxic or anoxic, but this would not explain why other fish and taxa did not normally inhabit this basin. Perhaps if the entire waters were anoxic and generally devoid of life then this would also explain the general absence or rarity of many fish and by extension the pterosaurs that would feed on them, but this is not apparently the case.

How this may align with the pterosaurs is therefore not clear at all. There is an abundance of pterosaurs that might not have frequented coastlines or flown over water, on top of the local avifauna (*Alcmonavis*, Rauhut, Tischlinger & Foth, 2019) and *Archaeopteryx*—Foth et al., 2025) that were not likely marine fliers, and the presence of at least some of the other reptiles implies this was not barren. This basin must, in some way, have been relatively devoid of aquatic life and thus was not frequented by aquatic feeding pterosaurs, but the presence of more terrestrial taxa and the bias for larger animals is harder to explain. More data is needed here, both on the actual composition of the fauna, and the taphonomy of the basin, the layers from which material was recovered, and the extent of any currents and water conditions to explain the taxa being found.

*Diversity*

**Figure 8:**
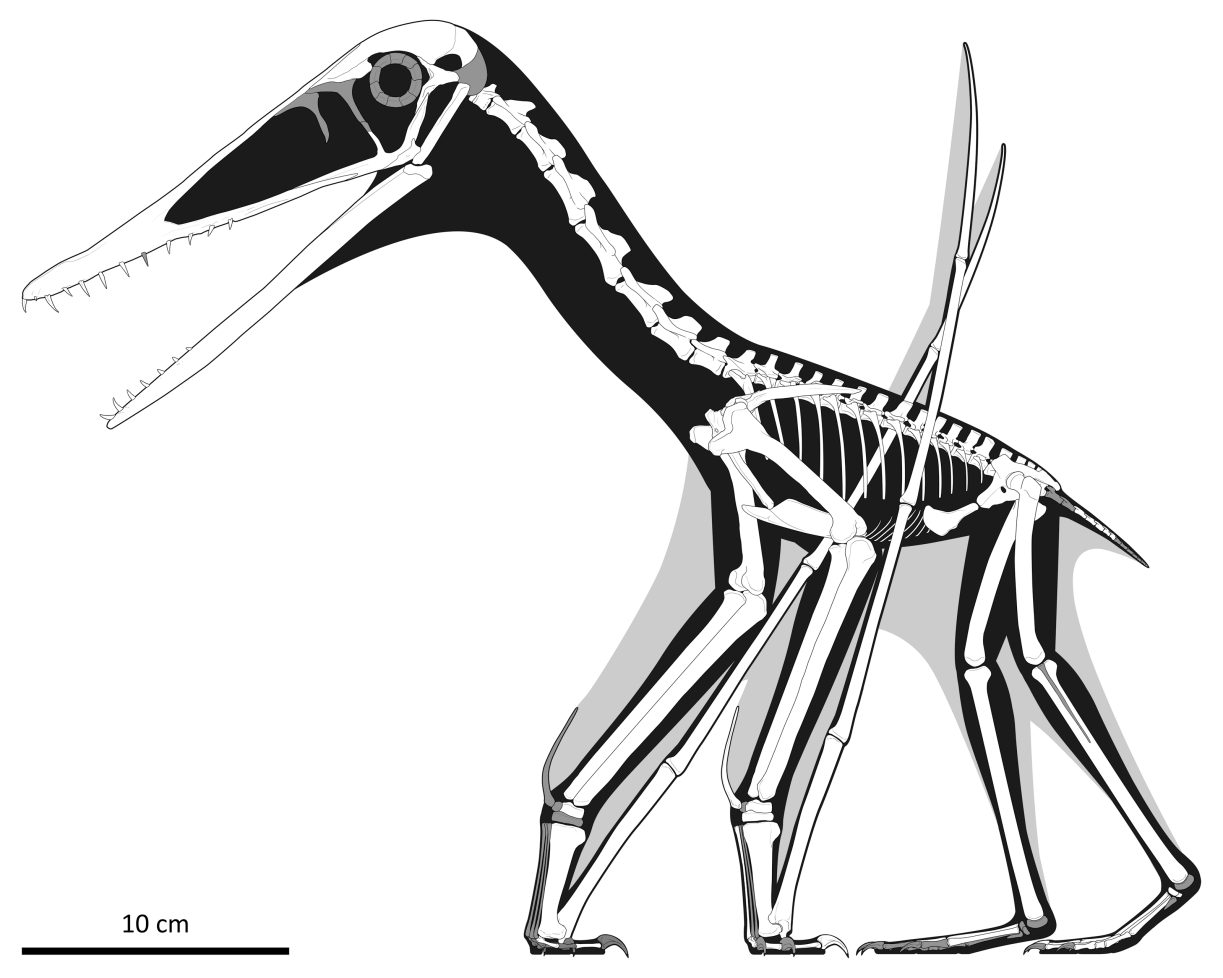
Skeletal reconstruction of *Lauropterusvitriolus* in left lateral view in a walking pose. Preserved elements are in white, with restored skeletal elements in dark grey. The main body outline is coloured black, with the restored wing membranes in pale grey. Scale bar is 10 cm. Artwork by Matt Dempsey, used with permission.

*Laueropterus* (Fig. 8) marks the fourth non-pterodactyloid monofenestratan pterosaur from the Mühlheim locality alongside *Skiphosoura*, *Makrodactylus*, and the ‘Rhamphodactylus’. The only other record of this grade in the region is *Propterodactylus*, in the much older Painten (Tischlinger & Frey, 2013; Spindler, 2024) locality, so these are not uniquely present in more recent beds, but they are clearly much more common here. Hundreds of pterosaur fossils have been recovered from the traditional Solnhofen beds to yield only *Propterodactylus*, but with four specimens of non-pterodactyloid monofenestratan in Mühlheim of perhaps less than a dozen pterosaurs that have been recovered (it is not known exactly how many have come from the quarry to date) this is very notable presence.

Although this is unusual in the number of specimens from this grade at one site, the distribution of pterosaurs in the region is often unbalanced with numerous representatives of a clade at one site that are rare nearby (Table 3). Assembling such a table is difficult given that for various historical specimens there are no details on the exact site they were found, or used dated names for formations, and so especially common taxa like *Pterodactylus* and *Rhamphorhynchus* may be present in other sites not listed here. Others are uncertain or unclear, *e.g.*, *Anurognathus* is given as ‘Solnhofen Limestone, Eichstätt’ (Wellnhofer, 1975) so could be Solnhofen Formation in the Eichstätt area, or in the Eichstätt locality specifically. Similarly, there is disagreement over the taxonomy and/or validity of some taxa listed with not all researchers agreeing on the list of genera given here. There is therefore uncertainty, but the general pattern and distribution should be informative.

**Table 3:** Distribution of pterosaur genera in Solnhofen region beds. The general layout is based on that of Fürsich et al. (2007). Localities are not in stratigraphic order and genera are listed for each site in alphabetical order.

**Age**	**Ammonite region**	**Formation**	**Malm Zeta**	**Locality**	**Genus**
Tithonian	Hybonotuum	Mörnsheim	3	Mühlheim	*Laueropterus* —this work *Makrodactylus* —Hone et al., 2025*Petrodactyle* —Hone et al., 2023*Scaphognathus* —Wellnhofer, 1975 ‘Rhamphodactylus’ —Rauhut, 2012*Rhamphorhynchus* —Arratia et al., 2015*Skiphosoura* —Hone et al., 2024
				Heinheim	
				Daiting	*Altmuelhopterus* —Wellnhofer, 1970*Ardeadactylus* —Bennett, 2013*Diopecepahlus* —Bennett, 2006
		Solnhofen (Altmühltal)	2b2a	Solnhofen	*Aerodactylus —*Vidovic & Martill, 2014*Aurorazhdarcho* —Bennett, 2013*Ctenochasma* —Wellnhofer, 1970*Diopecepahlus* —Bennett, 2006*Gnathosaurus*—Wellnhofer, 1970*Pterodactylus* —Bennett, 2013*Rhamphorhynchus* —Wellnhofer, 1975*Scaphognathus* —Bennett, 2014
			2a	Eichstätt	*Anurognathus* —Bennett, 2007*Ardeadactylus* —Bennett, 2013*Aurorazhdarcho* —Frey, Meyer & Tischlinger, 2011*Ctenochasma* —Wellnhofer, 1970*Cycnorhamphus* —Bennett, 1996*Germanodactylus* —Bennett, 2006*Gnathosaurus* —Wellnhofer, 1970*Pterodactylus* —Bennett, 2013*Rhamphorhynchus* —Wellnhofer, 1975
				Zandt Pfalzpaint	*Rhamphorhynchus* —Bennett, 1995
				Langenaltheim	*Rhamphorhynchus* —Bennett, 1995
Kimmeridigian	Becken	Rögling	1	Painten	*Cycnorhamphus* —Bennett, 2012*Propterodactylus* —Spindler, 2024*Pterodactylus* —Augustin et al., 2022
				Nusplingen	*Ardeadactylus* —Bennett, 2013*Cycnorhamphus* —Bennett, 1996*Rhamphorhynchus* —Wellnhofer, 1975
				Brunn	*Bellubrunnus* —Hone et al., 2012
				Schamhaupten	
		Torleite		Wattendorf	*Balaenognathus* —Martill et al., 2023
	Eudoxus/Pseudomutabilis	Treuchtlingen		Wattendorf	

As many as nine genera from a single formation are known, with as many as five or six ctenochasmatids together (Table 3). As such, finding a number of close relatives at Mörnsheim is perhaps to be expected. Indeed, this pattern can also be seen in other formations and localities where pterosaurs are common, with numerous species being found together (including some that are close relatives of one another) *e.g.*, the Yixian (Wang & Zhou, 2006), the Kem Kem (Longrich, Martill & Andres, 2018) and Daohugou (Sullivan et al., 2014)—the latter of which includes numerous darwinopterans. In short, where preservation is possible, pterosaurs are both common in terms of a high number of specimens being preserved, and diverse in terms of the taxa represented. This pattern is also seen with modern flying taxa around islands and lagoons with, for example, 15 species of cave-dwelling bats being found in a moderately sized island in the Philippines (Quibod et al., 2019) and 35 shorebird species from one island in India (Urfi, 2002) demonstrating that niche space can support numerous contemporaneous volant taxa, even at small sites.

Radiations of birds on isolated islands are a well-studied phenomenon, with considerable interest in the drivers behind these (Grant, 2001). One notable feature is that this may come primarily from feeding adaptations (*e.g.*, see Freed, Conant & Fleischer, 1987 on Hawaiian honeycreepers). However, although the Solnhofen region is generally understood to be an archipelago with various reefs around it, the islands are only c. 60 km from the mainland (Wellnhofer, 2009, his Fig 3.2). As such, they are nothing like as isolated as *e.g.*, the Galapagos or Hawaii, and are much more like the distance of many Mediterranean islands from the mainland. As a result, with pterosaurs being volant, such short distances were probably relatively easy to cross and it is likely that these animals were present on the mainland as well, and that they were not uniquely adapted to any given island or part of archipelago.

Even so, it is notable that these non-pterodactyloid monofenestratan pterosaurs are not all from the same horizon, and so it is not known if they even overlapped in time. Although *Makrodactylus* and the ‘Rhamphodactylus’ are apparently from the same horizon (Hone et al., 2025), others are vertically well separated. *Skiphosoura* is recorded 85 m below these taxa (Hone et al., 2024), though given the depth of the quarry this may be an error in recording from the GPS (since the quarry does not appear to be that deep), but they are clearly at least substantially separated and could have potentially been tens or hundreds of thousands of years apart. As such, between the different times and possible changes to local faunas, season variability, migrants, perhaps none of these taxa were ever contemporaneous in the ecosystem, let alone all of them. The consistency of the preservation of specimens in different layers does suggest at least that conditions were generally similar in the formation throughout the period of deposition. However, even if they did all overlap, there’s no *a priori* reason to think that multiple, closely related species could not be supported given what we see elsewhere in the region and beyond, and in modern analogues.

## Conclusions

It is somewhat ironic that after multiple (and reasonable) taxonomic revisions that have dramatically reduced the numbers of taxa from southern Germany (*e.g.*, Wellnhofer, 1975, Bennett, 1995; Bennett, 1996) renewed excavations are now rapidly increasing the number of pterosaurs from the Solnhofen region lithographic limestones. Three new taxa have been described from Mörnsheim with other new finds in Wattendorf (Martill et al., 2023), Brunn (Hone et al., 2012) and even in the classic Solnhofen beds (Frey, Meyer & Tischlinger, 2011). In short, pterosaurs were likely generally both common and diverse in many, even most, ecosystems and the enormous taphonomic filter of their fragile bones (in addition to destruction by carnivores) means that they are generally rare, except in areas of exceptional preservation. Given the quality of preservation at Mörnsheim and the number of specimens found in a relatively short space of time, it is likely that many more specimens and pterosaur taxa remain to be uncovered here.

## Institutional Abbreviations

BSPG: Bavarian State Collection for Palaeontology and Geology, Munich, Germany
LF: Lauer Foundation for Paleontology, Science and Education, Wheaton, Illinois, USA
NHM-UK: Natural History Museum, London, UK

## Additional Information and Declarations

### Data Availability

The following information was supplied regarding data availability: The specimen is permanently held with the Lauer Foundation for Paleontology, Science and Education in the USA: LF 6268.

### New Species Registration

The following information was supplied regarding the registration of a newly described species: *Laueropterus vitriolus*, Pterosauria Publication LSID: urn:lsid:zoobank.org:pub:4E0CF077-2DA5-451D-934E-103801608A36 Genus LSID: urn:lsid:zoobank.org:pub:ce3a64d1-4698-4d57-b2fb-41a1edd2af38 Species LSID: urn:lsid:zoobank.org:pub:E6B29691-795C-4B04-8DFE-14EDDFF13B0A
